# From Innovation to Complication: A Case Report and Review on Immune-Related Colitis Induced by ICIs

**DOI:** 10.3390/ph18081211

**Published:** 2025-08-15

**Authors:** Huibo Li, Yumiao Pan, Wenzheng Liu, Hejun Zhang, Xueli Tian, Rongsheng Zhao, Yi Zhun Zhu

**Affiliations:** 1 Department of Pharmacy, Peking University Third Hospital, Beijing 100191, China; lihuibo@bjmu.edu.cn (H.L.); pym1996@126.com (Y.P.); 2 State Key Laboratory of Quality Research in Chinese Medicine, School of Pharmacy, Macau University of Science and Technology, Macau SAR 999078, China; 3 Department of Pharmacy, Peking University Third Hospital Qinhuangdao Hospital, Qinhuangdao 066000, China; 4 Department of Gastroenterology, Peking University Third Hospital, Beijing 100191, China; dliu@hsc.pku.edu.cn (W.L.); hjzhang99@126.com (H.Z.); tiantian1188_163@163.com (X.T.); 5 Shanghai Key Laboratory of Bioactive Small Molecules, Department of Pharmacology, School of Pharmacy, Fudan University, Shanghai 200437, China

**Keywords:** immune checkpoint inhibitors, diarrhea, colitis, sintilimab, immune-related adverse events

## Abstract

Immune checkpoint inhibitors (ICIs) have revolutionized cancer therapy by providing durable responses and a favorable safety profile, ushering in a new era of tumor immunotherapy. However, immune-related adverse events (irAEs) remain a significant clinical challenge. Among these, gastrointestinal irAEs, especially immune-related colitis (ir-colitis), can lead to serious complications if not promptly recognized and managed. Here, we present a case of grade 3 ir-colitis induced by the programmed cell death protein 1 (PD-1) inhibitor sintilimab in a 68-year-old woman with endometrial cancer. The patient developed severe acute diarrhea following ICI administration, which progressed despite initial antidiarrheal and antimicrobial treatments. A multidisciplinary team (MDT) involving gastroenterologists, oncologists, a pathologist, and a clinical pharmacist confirmed the diagnosis and implemented high-dose corticosteroid therapy, yielding significant clinical improvement. Importantly, this report highlights the mechanistic link between PD-1 blockade and ir-colitis pathogenesis, focusing on the dysregulation of the mucosal immune environment and its role in triggering colonic injury. In addition to the case description, we provide a comprehensive review of the literature and clinical guidelines, discussing risk factors, diagnostic approaches, therapeutic strategies, and long-term monitoring. By integrating insights from pharmacology, immunology, and clinical practice, this work emphasizes the importance of early detection, patient education, and MDT collaboration for optimizing therapeutic outcomes and advancing the understanding of ir-colitis in the context of ICI therapy.

## 1. Introduction

The advent of immune checkpoint inhibitors (ICIs) has revolutionized cancer therapy, significantly improving survival outcomes and quality of life in patients with advanced malignancies [1,2]. Despite their clinical benefits, ICIs are associated with a broad spectrum of immune-related adverse events (irAEs), most commonly affecting the skin, thyroid, gastrointestinal tract, liver, and pituitary gland [3]. Among these, immune-related colitis (ir-colitis) presents a particular concern. While the overall incidence of ir-colitis is estimated at approximately 11%, a meta-analysis of PD-1 inhibitor monotherapy reported a much lower incidence of around 0.9%, with grade 3–4 events occurring in only about 0.6% of patients [4,5]. Clinically, ir-colitis typically presents with diarrhea (with or without mucus or bloody stools), abdominal pain, and weight loss, with endoscopic findings including ulceration, erythema, and loss of vascular architecture [6,7]. However, its insidious onset and nonspecific gastrointestinal symptoms often overlap with infectious or chemotherapy-induced colitis, contributing to delayed diagnosis and suboptimal management. This delay increases the risk of refractory inflammation, impairs quality of life, and imposes a substantial healthcare burden.

Sintilimab, a novel PD-1 inhibitor, has demonstrated potent antitumor efficacy across multiple solid tumors. Nevertheless, it is also associated with various irAEs, including immune-mediated diabetes mellitus [8], thyroid dysfunction [9], dermatologic toxicities [10], and urologic adverse events [11]. Notably, sintilimab-induced colitis is rarely reported, with an estimated incidence of <1%, and severe (grade ≥ 3) cases occurring in fewer than 0.5% of treated patients [12]. This underreporting, combined with the lack of standardized diagnostic and therapeutic protocols, may contribute to delayed clinical recognition, leading to treatment interruptions, disease progression, and diminished patient autonomy.

This study aims to comprehensively elucidate the pathogenesis and clinical pharmacotherapeutic management of sintilimab-induced colitis in an elderly patient with advanced endometrial cancer. By synthesizing recent advances in immunology and pharmacology, we provide a detailed exploration of the underlying immunopathological mechanisms driving ir-colitis, with particular emphasis on the dysregulation of intestinal immunity induced by ICIs. Employing an MDT approach, we highlight the essential role of clinical pharmacists in the early identification, risk stratification, and ongoing pharmacovigilance of immune-related adverse events. Through evidence-based strategies, this report concentrates on optimizing patient safety and therapeutic outcomes, thereby contributing practical insights toward the precision management of irAEs in oncology practice.

## 2. Case Report

### 2.1. The Patient’s History and Clinical Presentation

A 68-year-old woman (156 cm, 51 kg, BMI 20.96 kg/m^2^) with stage IIIA moderately to poorly differentiated endometrial adenocarcinoma underwent laparoscopic radical surgery in September 2018. Following the surgery, she received 20 cycles of chemotherapy with paclitaxel (270 mg) and carboplatin (600 mg), experiencing nausea and vomiting but no abdominal pain or diarrhea.

In August 2023, tumor recurrence was documented, leading to the initiation of camrelizumab (200 mg) plus chemotherapy. Ten days post-administration, the patient developed grade 1 immune-related diarrhea, characterized by approximately three episodes daily and a fecal volume of about 400 mL, which resolved spontaneously without intervention. We hypothesized that this adverse effect was related to camrelizumab, a humanized anti-PD-1 monoclonal antibody characterized by a moderately strong PD-1 binding affinity and an engineered Fc region that retains partial effector function. Camrelizumab predominantly targets the FG loop of the PD-1 receptor and may cross-react with adjacent epitopes such as the CC’ loop, potentially leading to broader T-cell activation and increased intestinal immune perturbation. In contrast, sintilimab demonstrates higher PD-1 binding affinity, targeting both the FG and CC’ loops more precisely, and incorporates the stabilizing S228P mutation in its Fc region, minimizing Fcγ receptor-mediated effector functions. These molecular characteristics contribute to a more controlled T-cell activation profile and a lower incidence of immune-mediated colitis [13,14]. Given the patient’s prior gastrointestinal symptoms, sintilimab (200 mg) was selected for the subsequent immunotherapy cycle (14 September 2023) to reduce the risk of immune-related enterocolitis while maintaining antitumor efficacy.

However, 40 days after sintilimab administration (23 October 2023), the patient developed worsening diarrhea of uncertain cause. Initially, she experienced yellow, formed stools (5–10 times per day), which progressed over the next six days to yellow, watery stools (10–15 times per day) of variable volume, accompanied by intermittent fever (up to 37.7 °C) and periumbilical colicky pain (Visual Analog Scale score of 3). Treatment with montmorillonite powder and Bacillus licheniformis provided no relief.

Ten days later, the patient presented with purulent and bloody stools (without mucus) and persistent abdominal pain. Levofloxacin and Montmorillonite Powder for five days at an external facility failed to yield significant improvement. Seventeen days later, her diarrhea worsened, characterized by watery, bloody stools with a blood volume of approximately 10 mL, occurring 10–16 times per day, and a total daily volume ranging from 1000 to 1500 mL. The patient experienced intermittent diarrhea for about 20 days, during which anti-diarrheal agents and empirical antibiotic therapy proved ineffective, and her condition continued to worsen. The patient had no history of long-term use of aspirin or other nonsteroidal anti-inflammatory drugs (NSAIDs). The patient’s surgical and prior medication history is summarized in Supplementary Table S1.

### 2.2. The Patient’s Treatment Process

On 13 November 2023, the patient was admitted for further evaluation and management. Upon admission, her vital signs were stable (temperature: 36.4 °C, pulse: 68 bpm, respiratory rate: 18 breaths/min, and blood pressure: 118/64 mmHg), but the physical examination revealed hyperactive bowel sounds (6 beats/min). Laboratory investigations demonstrated markedly elevated fecal calprotectin (183.23 μg/g, reference range 0–15 μg/g), C-reactive protein (18 mg/L, reference range 0–10 mg/L), IL-8 (36.65 pg/mL), IL-9 (1.81 pg/mL), and G-CSF (12.38 pg/mL), indicative of active intestinal inflammation, while inflammatory markers, including white blood cell count, procalcitonin (PCT), and cytomegalovirus, remained within normal limits. This may be attributed to the localized nature of ir-colitis, which predominantly involves the gastrointestinal tract and often fails to trigger a systemic inflammatory response [15,16]. Significant decrease in lymphocyte subset counts, including CD4/CD8 Ratio (0.65), CD16/CD56 (66.51), CD3+ T Lymphocyte Absolute Count (272.43 cells/μL), CD4+ T Helper Cell Absolute Count (105.34 cells/μL), CD8+ Cytotoxic T Cell Absolute Count (161.19 cells/μL), and CD19+ B Lymphocyte Absolute Count (68.31 cells/μL), suggests impaired immune competence and potentially point to the presence of immune-related pathologies. Tests for Clostridium difficile toxins and antigens, antinuclear antibodies (ANA), and T-SPOT were negative, and the patient had no history of lactose intolerance. Whole blood biochemical parameters, electrolyte levels before and during treatment, cytokine, lymphocyte subset counts, cytomegalovirus (CMV), Epstein–Barr virus (EBV), and PCT levels are presented in the Supplementary Tables S2–S5 and Figures S1–S3.

**Figure 1:**
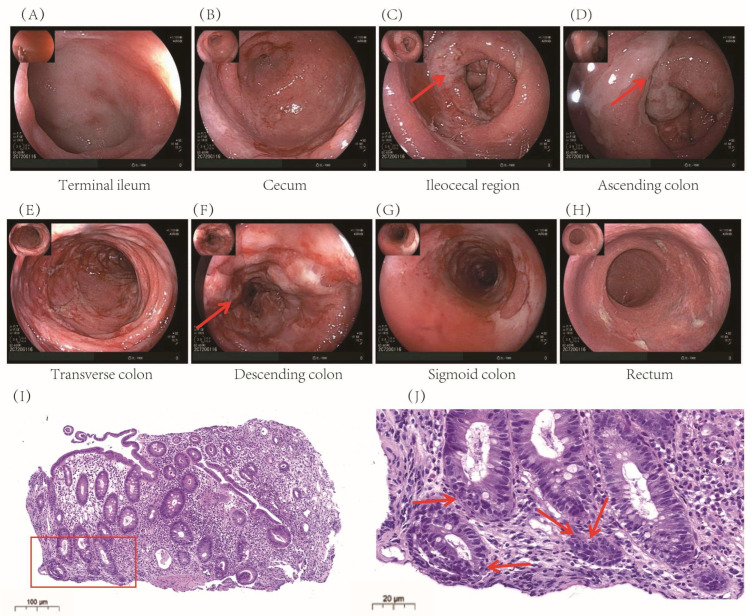
Electronic colonoscopy and pathological images of the patient after Sintilimab. (**A**–**H**): Colonoscopy revealed diffuse intestinal involvement from the terminal ileum to the rectum. Specifically, (**C**) shows diffuse mucosal hyperemia and edema (indicated by arrows); (**D**) shows the presence of white pseudomembranes (indicated by arrows); and (**F**) reveals ulcers and herpetiform lesions (indicated by arrows). (**I**) Hematoxylin and eosin-stained sections of crypt epithelium and a significant reduction in cup cells in colonic tissues, evident in cryptitis, crypt abscesses, crypt atrophy, and structural disturbances in the arrangement of crypt tissues, the area marked with a red box is enlarged in panel (**J**) to provide a clearer view of the colonic mucosal lesions; and (**J**) highlights scattered apoptotic bodies within the lamina propria and crypt epithelium (indicated by arrows), suggestive of immune-mediated mucosal injury.

Colonoscopy revealed extensive mucosal edema from the terminal ileum to the colon, along with spasm, pseudomembrane formation, and friable mucosa prone to hemorrhage (Figure 1A–H). Histopathological examination confirmed significant colonic inflammation, characterized by mucosal erosion, tissue necrosis, and inflammatory exudates, findings consistent with immune-related colitis (Figure 1I, J).

The patient was initially treated with intravenous cefoperazone (1 g, 3 times daily) and metronidazole (0.5 g, 2 times daily) for broad-spectrum antibiotic coverage, oral montmorillonite powder (3 g, 3 times daily) for symptomatic control of diarrhea, and bifidobacterium triple viable capsules (840 mg, 2 times daily) to regulate the gut microbiota. Despite treatment, the patient experienced persistent diarrhea (15–20 episodes/day). On 17 November, intravenous methylprednisolone (40 mg/day) was initiated; however, after 3 days, stool frequency remained at 10–20 times/day.

Given the refractory nature of the symptoms, a multidisciplinary team (MDT) consultation, including gastroenterologists, oncologists, a pathologist, and a clinical pharmacist, was conducted to reassess the patient’s clinical history, disease progression, and differential diagnosis. From the perspective of pharmacotherapy management, the clinical pharmacist reviewed the patient’s medication history, evaluated the clinical trajectory, and strongly suspected grade 3 ir-colitis. Based on the National Comprehensive Cancer Network (NCCN) Guidelines for Management of irAEs, the pharmacist recommended escalating methylprednisolone to 1–2 mg/kg/day [17]. Consequently, the dose was increased to 40 mg IV BID (total 80 mg/day) on November 20, with a taper planned over 4–6 weeks. The patient was maintained on a nil per os (NPO) status and received nutritional support with a caloric target of 1560 kcal/day and protein intake of 1–1.5 g/kg/day. Nutritional supplementation consisted of 27 scoops per day of oral enteral nutrition powder, IV medium- and long-chain triglyceride (MCT/LCT) fat emulsion (250 mL), and a compound amino acid solution (18AA-7). Following nutritional support and disease control, the patient’s prealbumin (PA), albumin (ALB), and total protein (TP) levels showed a gradual upward trend, reflecting improvement in both nutritional status and systemic recovery.

**Figure 2:**
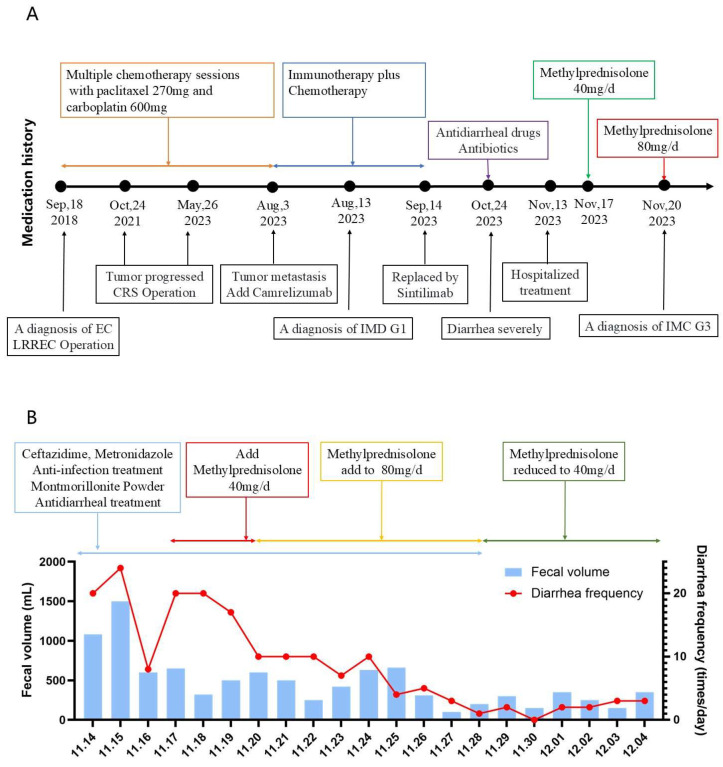
Correlation between diarrhea severity and treatment timeline. (**A**) Schematic timeline of key therapeutic interventions and clinical events from 2018 to 2023. Key clinical milestones include: initial diagnosis of endometrial carcinoma (EC) and cytoreductive surgery (CRS) (September 2018); disease progression (October 2021); metastatic spread and initiation of camrelizumab (May 2023); transition to sintilimab (3 August 2023); hospitalization (13 August 2023); diagnosis of grade 1 immune-mediated diarrhea/colitis (IMD G1) (14 September 2023); and subsequent diagnosis of grade 3 immune-mediated colitis (IMC G3) (24 October 2023). Detailed management of IMC G3 is illustrated in panel. (**B**) Quantification of diarrheal symptoms (fecal volume and frequency) in hospital. Anti-infectives (ceftazidime, metronidazole), smectite (antidiarrheal), and methylprednisolone dose adjustments (40 mg/d on 13 Nov → 80 mg/d on 17 Nov → 40 mg/d on 20 Nov).

Given the expected exposure to high-dose corticosteroids (≥20 mg/day prednisone equivalent for ≥4 weeks), Pneumocystis jirovecii pneumonia (PJP) prophylaxis was initiated with oral co-trimoxazole (800 mg twice daily), as advised by the clinical pharmacist. Remarkably, 1 day after steroid escalation, the patient’s stool frequency decreased to <10 times/day, and by day 6, to 0–5 soft, formed stools/day (total volume: 0–500 mL/day). By 5 December 2023, the diarrhea had fully resolved, and the patient’s vital signs remained stable. Following a corticosteroid taper, the patient was discharged. The patient’s medication history and treatment responses are summarized in Figure 2.

Due to the severity of ir-colitis, ICI therapy was discontinued in accordance with guidelines. Reinitiation was deferred until toxicity resolved to grade ≤ 1. At the 6 week post-discharge follow-up, the patient reported 3–4 episodes of diarrhea per day, characterized by yellow, pasty stools, with fecal occult blood testing (FOBT) remaining positive. At the 14 week follow-up, the frequency of diarrhea had decreased to 1–2 times per day, with yellow, soft stools; however, the FOBT continued to be positive. At the 1 year follow-up, the patient reported 0–2 normal bowel movements per day, a negative FOBT, and no recurrence of colitis symptoms.

## 3. Discussion

### 3.1. Assessment of Adverse Drug Reactions

The patient developed severe, progressive diarrhea on 23 October 2023, which was not observed during previous chemotherapy cycles involving paclitaxel liposomes and carboplatin, agents known to disrupt gut microbiota and sensitize intestinal mucosa. No significant changes were observed in inflammatory markers such as PCT and WBC; both EBV and CMV tests were negative, further excluding infectious etiologies. Immunological evaluation revealed marked lymphopenia, with reductions observed across multiple lymphocyte subsets, including CD4+ T cells, CD8+ T cells, and natural killer (NK) cells. This lymphocyte depletion, coupled with elevated levels of proinflammatory cytokines (IL-8, IL-9, G-CSF), indicated profound immune dysregulation consistent with an immune-related adverse event. Notably, symptoms emerged 40 days after the initiation of sintilimab, aligning well with the reported onset times of sintilimab-induced colitis, which range from 6 to 374 days, with a median onset of 112 days [18]. Based on this timeframe, sintilimab was considered the most probable causative agent.

To quantitatively assess the causality of sintilimab-induced colitis, we applied the Naranjo Adverse Drug Reaction Probability Scale, a widely accepted tool for evaluating drug-related adverse events [19,20]. The patient’s total score was 6, indicating a “probable” association between sintilimab and the adverse event (details in Supplementary Table S6). The clinical pharmacist diagnosed the patient with grade 3 immune-related colitis (ir-colitis) induced by sintilimab, which was further supported by the prompt resolution of diarrhea following the discontinuation of sintilimab and methylprednisolone therapy (40 mg IV, twice daily). Of note, the patient had previously developed grade 1 self-limiting diarrhea after receiving camrelizumab on 3 August 2023, suggesting a predisposition to ICI-induced gastrointestinal toxicity.

### 3.2. Mechanisms of Immune-Related Colitis

Sintilimab is a fully humanized IgG4 monoclonal antibody that targets PD-1 with high specificity and affinity for its FG loop region [21]. Under physiological conditions, engagement of PD-1 by its ligand programmed death-ligand 1 (PD-L1) negatively regulates T cell receptor (TCR)-mediated signaling pathways, suppressing the expression of proinflammatory cytokines, including IL-2 and IFN-γ [22]. These signaling cascades are essential for maintaining peripheral immune tolerance and preventing autoreactive immune responses. Although the fully human structure of sintilimab is associated with a lower risk of irAEs [21], its potent immunostimulatory activity can override immune homeostasis, increasing the susceptibility to ir-colitis.

PD-1 blockade enhances the proliferation and activation of effector T cells (Teff) while concurrently impairing the suppressive function of regulatory T cells (Tregs) [17]. In patients with ir-colitis, histological analyses reveal significant infiltration of CD8^+^ tissue-resident memory T cells (Trm) in the colonic mucosa, which further differentiate into cytotoxic T lymphocytes (CTLs) capable of secreting high levels of IFN-γ and TNF-α [18]. At the same time, Tregs dysfunction leads to diminished secretion of essential immunosuppressive cytokines, including IL-10 and TGF-β. This imbalance favors the expansion of proinflammatory Th1 and Th17 cells, resulting in increased levels of IL-17 in the gut mucosa and upregulation of chemokines such as TNF-α, IL-6, CXCL9, and CXCL10 [19,20]. These inflammatory mediators facilitate the recruitment of additional T cells and neutrophils to inflamed tissues, amplifying the mucosal immune response. In some instances, Tregs undergo phenotypic plasticity, acquiring an IFN-γ–producing Th1-like profile [21], thereby losing their immunosuppressive capacity and further promoting mucosal inflammation.

Histopathologically, one of the defining features of ir-colitis is the increased rate of apoptosis in colonic crypt epithelial cells [23]. This apoptosis is primarily mediated by activated CD8^+^ CTLs through both extrinsic and intrinsic pathways, including Fas/FasL signaling leading to caspase-8–dependent extrinsic apoptosis, and the perforin-granzyme B axis, which induces epithelial cell lysis and caspase-3–dependent intrinsic apoptosis [24]. These processes contribute to crypt architectural distortion and disruption of the epithelial barrier. Additionally, PD-1 inhibitors may induce the production of autoantibodies targeting the integrin αVβ6, an epithelial adhesion molecule, thereby further impairing epithelial integrity and exacerbating tissue damage [25,26].

As the intestinal barrier becomes increasingly compromised, microbial translocation of luminal bacteria and their metabolites into the lamina propria ensues, activating innate immune cells and promoting diffuse mucosal inflammation [27]. Clinical studies have demonstrated that patients with ICI-induced colitis exhibit significantly reduced gut microbial diversity [28]. In particular, a marked decrease in the relative abundance of *Faecalibacterium prausnitzii* and *Bacteroides fragilis* has been consistently observed [29]. *F. prausnitzii* is known for its critical role in maintaining colonic mucosal integrity, primarily through the production of butyrate and other short-chain fatty acids that support epithelial barrier function and modulate local immune responses [30,31]. Meanwhile, *B. fragilis* exerts potent anti-inflammatory effects by inducing IL-10 production via its capsular polysaccharide A [32], thereby contributing to the attenuation of mucosal inflammation and the preservation of immune homeostasis.

**Figure 3:**
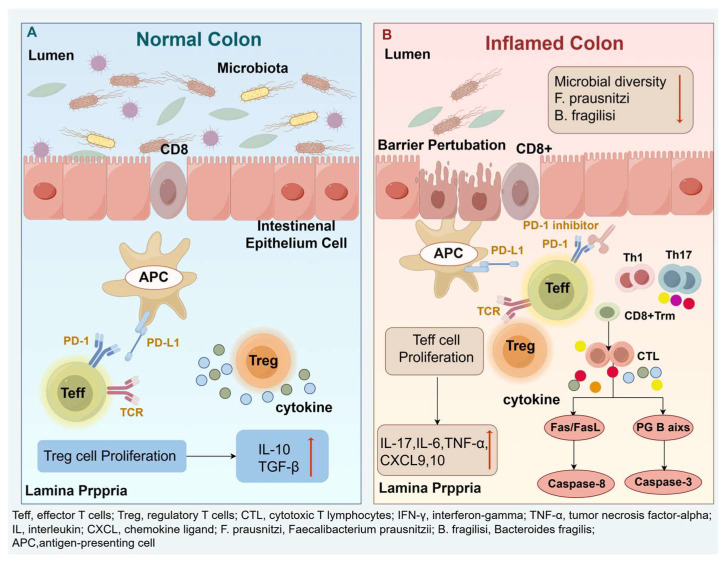
Mechanisms of ir-colitis: (**A**) Under physiological conditions, the migration of T lymphocytes to the intestinal mucosa is tightly regulated, with preserved epithelial integrity and a diverse, balanced gut microbiota. (**B**) PD-1 inhibition disrupts this equilibrium by enhancing the activation and proliferation of Teff while impairing Treg function. In the colonic mucosa, massive infiltration and differentiation of CD8^+^ T cells into CTLs results in the excessive release of proinflammatory cytokines, such as IFN-γ and TNF-α. Concurrently, activation of Th1 and Th17 cells amplifies local inflammation through the production of IL-17, TNF-α, IL-6, CXCL9, and CXCL10. Activated CTLs induce epithelial cell apoptosis via Fas/FasL signaling and the perforin–granzyme B axis, leading to mucosal barrier disruption. The consequent increase in epithelial permeability facilitates microbial translocation into the lamina propria, where innate immune activation further exacerbates mucosal inflammation. These synergistic processes culminate in the development of ir-colitis.

The combination of immune dysregulation, epithelial damage, and microbial translocation results in impaired absorptive function, increased vascular permeability, and microvascular injury [33]. Clinically, these alterations manifest as acute watery diarrhea, often accompanied by hematochezia, mucus-containing stools, and crampy abdominal pain—hallmarks of sintilimab-induced colitis (Figure 3. Mechanisms of ir-colitis provides a schematic overview of the pathophysiology, including checkpoint blockade, T-cell dysregulation, epithelial damage, and microbiota-associated factors).

### 3.3. Risk Factors for ir-Colitis Development

The risk of ir-colitis associated with PD-1 blockade is influenced by the specific agent used, treatment regimen, underlying malignancy, and patient-level comorbidities. Combination therapy with PD-1 and CTLA-4 inhibitors significantly increases the risk, with an overall incidence of up to 30% [34]. In a real-world cohort of 362 lung cancer patients, the incidence of ir-colitis varied across PD-1 inhibitors: sintilimab (3.1%), camrelizumab (3.2%), pembrolizumab (5.0%), and tislelizumab (3.6%). Grade 3–4 colitis occurred in 1.0% and 0.8% of patients treated with camrelizumab and pembrolizumab, respectively, while no high-grade events were reported in the sintilimab group [35]. The risk appears to be dose-dependent, with ir-colitis most commonly emerging after the third treatment cycle [36].

Additional risk factors include tumor biology and patient characteristics. Patients with melanoma have higher ir-colitis rates compared to those with non-small cell lung cancer (NSCLC) or renal cell carcinoma, likely due to distinct tumor-immune microenvironment interactions between the gut microbiome and the tumor-immune microenvironment [37]. Individuals with pre-existing autoimmune diseases, particularly inflammatory bowel disease (IBD), face heightened susceptibility, with colitis incidence rates reaching 40% [38,39]. Gender differences have also been observed: data from the FDA Adverse Event Reporting System indicate that 53.5% of irritable colitis cases occurred in males versus 33.2% in females, although this disparity warrants further study [40].

### 3.4. Therapeutic Management of ir-Colitis

**Figure 4:**
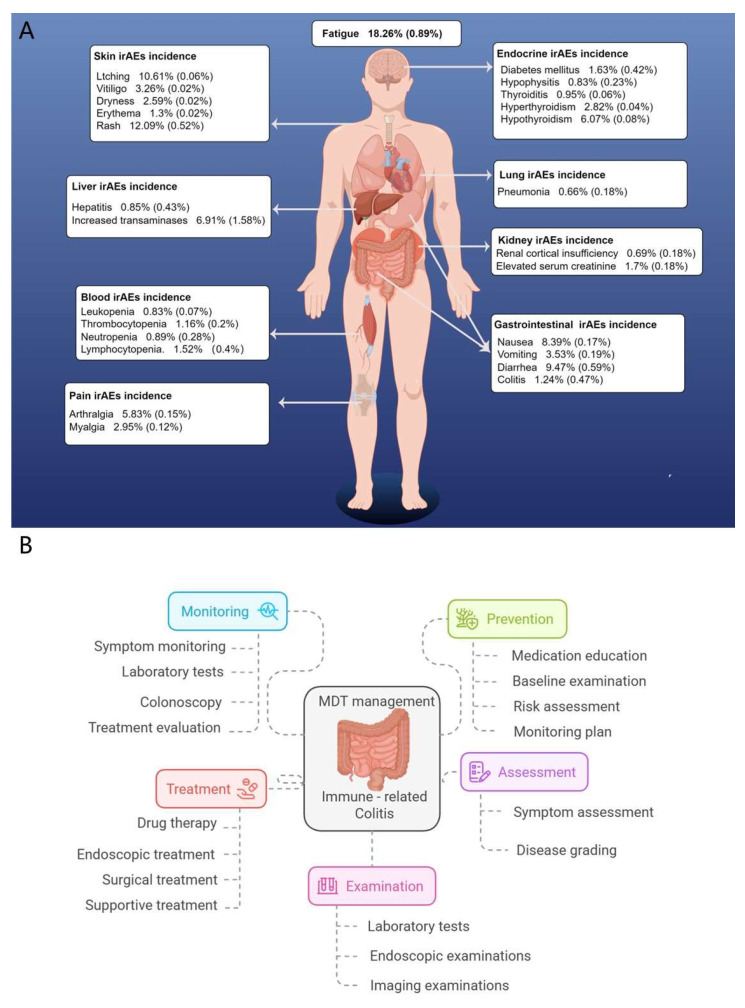
Incidence of irAEs to PD-1 inhibitors and multidisciplinary management of ir-colitis. (**A**) Overview of common immune-related adverse events (irAEs) associated with immune checkpoint inhibitors (ICIs), categorized by organ system and reported incidence. Fatigue is the most frequently observed irAE, followed by dermatologic, gastrointestinal, hepatic, endocrine, pulmonary, renal, and hematologic toxicities. Data represents overall and high-grade (≥Grade 3) incidence rates. (**B**) Multidisciplinary team (MDT) management framework for immune-related colitis. Key components include symptom monitoring, preventive strategies, early assessment, diagnostic examinations, and multimodal treatment approaches such as pharmacologic, endoscopic, or surgical interventions.

Gastrointestinal irAEs are among the most common toxicities associated with PD-1 inhibitors, second only to dermatologic events, and more frequent than endocrine toxicities [41]. However, their nonspecific presentation—diarrhea, abdominal pain—can lead to diagnostic delays. Given these challenges, an early MDT intervention is essential, involving oncologists, gastroenterologists, radiologists, pathologists, pharmacists, nutritionists, and psychologists to deliver comprehensive care. Timely identification and intervention not only improve clinical outcomes but also enhance patients’ quality of life and treatment adherence (see Figure 4).

Management strategies are guided by evidence-based recommendations from major oncology societies, including the Society for Immunotherapy of Cancer (SITC), American Society of Clinical Oncology (ASCO), National Comprehensive Cancer Network (NCCN), European Society for Medical Oncology (ESMO), and Chinese Society of Clinical Oncology (CSCO) [17,42,43,44,45]. A comparative overview is presented in Supplementary Table S7.

For grade 1 ir-colitis, symptomatic management with loperamide, atropine, dietary modifications, and possibly mesalazine is sufficient [17,42,43]. Immunotherapy can typically be continued. In grade 2 ir-colitis, infections should be ruled out, and topical budesonide (9 mg/day) is the preferred initial treatment by NCCN as its enteric-coated formulation allows targeted release in the terminal ileum and right colon, making it theoretically advantageous for localized mucosal inflammation while minimizing systemic corticosteroid exposure, particularly in cases with mild to moderate symptoms [17]. A retrospective study by Machado et al. demonstrated that budesonide achieves remission rates comparable to systemic corticosteroids [46]. However, budesonide has significant limitations. Its efficacy in moderate to severe or extensive colitis remains uncertain, and it is generally not recommended as monotherapy for grade 2 ir-colitis in both international guidelines and expert consensus in China [42,43,44,45]. Initial management still involves the oral corticosteroids, typically prednisone or methylprednisolone at a dose of 1 mg/kg/day. If symptoms fail to improve within 3–5 days, escalation to intravenous corticosteroids or biologics may be necessary.

For grade ≥ 3 ir-colitis, immediate discontinuation of ICIs is mandatory. Patients require hospitalization, continuous monitoring, and urgent IV corticosteroid therapy (methylprednisolone 1–2 mg/kg/day). For steroid-refractory cases, occurring in up to 60% of patients, escalation to biologics is necessary. Infliximab (5 mg/kg) [47] or vedolizumab (α4β7 integrin antagonist) are first-line agents [48]. Additionally, ustekinumab (an IL-12/IL-23 inhibitor) is also effective in select refractory cases [49].

Emerging treatment strategies include Janus kinase (JAK) inhibitors and microbiota-targeted interventions. Tofacitinib (10 mg twice daily) has achieved a 75% clinical remission rate in steroid-refractory IBD [50,51], while upadacitinib (45 mg/day) has demonstrated rapid efficacy in severe cases [52]. Fecal microbiota transplantation (FMT) has demonstrated a 58% clinical remission rate within four weeks and is being investigated as a salvage therapy [53].

While novel biologics and small molecules offer hope, their use is limited by potential risks, including infection reactivation (e.g., hepatitis B, tuberculosis), systemic immunosuppression, and incomplete safety profiles. JAK inhibitors, in particular, show promise due to oral administration and rapid onset but require further validation. Future trials should evaluate the safety, efficacy, and durability of these interventions in oncologic populations [54].

## 4. Conclusions

Sintilimab-induced ir-colitis represents a serious but underrecognized complication of cancer immunotherapy, underscoring the need for timely diagnosis and evidence-based management. Advancing our understanding of its immunopathogenesis, refining risk stratification, and developing targeted therapeutic approaches will be pivotal for optimizing patient outcomes and maximizing the clinical benefits of PD-1 inhibitors. Future prospective studies are warranted to validate emerging treatments and to refine clinical guidelines for managing immune-mediated gastrointestinal toxicities.

## Data Availability

The original contributions presented in this study are included in the article/Supplementary Materials. Further inquiries can be directed at the corresponding authors.

## Supplementary Materials

The following supporting information can be downloaded at: https://www.mdpi.com/article/10.3390/ph18081211/s1, Table S1: Patient's surgical and prior medication history; Table S2: CBA Cytokine levels during hospitalization; Table S3: Lymphocyte subset counts levels during hospitalization; Table S4: The results of blood and fecal tests of the patient; Table S5: The results of blood and fecal tests of the patient; Table S6: Naranjo Adverse Drug Reaction Assessment Scale; Table S7: Specific therapeutic strategies for ir-colitis in various guidelines; Figure S1: PCT levels during hospitalization; Figure S2: Serum ALB, TP, and PA levels during hospitalization and at 18-month follow-up; Figure S3: Serum Na+, K+ levels during hospitalization and at 18-month follow-up.
