# Supplementary material for: From Innovation to Complication: A Case Report and Review on Immune-Related Colitis Induced by ICIs
Source: Pharmaceuticals (Basel). 2025 Aug 15;18(8):1211. doi: 10.3390/ph18081211 (PMC12389329; doi:10.3390/ph18081211)
Supplement: Supplementary file 1 [file pharmaceuticals-18-01211-s001.zip › pharmaceuticals-3737340-supplementary.pdf]

pharmaceuticals

Case Report

Supplementary Material

Supplementary Tables

Table S1. Patient's surgical and prior medication history.

Time	Surgery and Treatment	Adverse Reactions
9/18/2018	Laparoscopic radical endometrial cancer surgery	None
10/9/2018-10/21/2021	Paclitaxel 270 mg + Carboplatin 600 mg Chemotherapy 3 times	Nausea, vomiting
10/24/2021	Tumor subtraction	None
10/24/2021-5/26/2023	Paclitaxel 270 mg + carboplatin 600 mg chemotherapy 10 times	Nausea
5/26/2023	Tumor subtraction	None
8/3/2023	Paclitaxel 270 mg + Carboplatin 600 mg + Carelizumab 200mg (Humanized anti-PD-1 monoclonal antibody)	G1 Immune-related diarrhea
9/14/2023	Paclitaxel 270 mg + Carboplatin 600 mg + Sindilizumab 200 mg (Recombinant fully humanized anti-PD-1 monoclonal antibody)	G3 Immune-related colitis

Table S2. CBA Cytokine levels during hospitalization. The red text is used to emphasize values above the normal range.

Test Item Name	Test Result (pg/ml)	Reference Range (pg/ml)
Interleukin-1β (IL-1β)	0.03	≤12.4
Interleukin-2 (IL-2)	1.00	≤7.5
Interleukin-4 (IL-4)	0.99	≤8.56
Interleukin-5 (IL-5)	1.26	≤3.1
Interleukin-6 (IL-6)	4.42	≤5.4
Interleukin-8 (IL-8)	**36.65**	≤20.6
Interleukin-9 (IL-9)	**1.81**	≤1.54
Interleukin-10 (IL-10)	7.41	≤12.9
Interleukin-12p70 (IL-12p70)	0.41	≤3.4
Interleukin-17 (IL-17)	3.42	≤21.4
Interferon-α (IFN-α)	2.42	≤8.5
Interferon-γ (IFN-γ)	22.17	≤23.1

Tumor Necrosis Factor-α (TNF-α)	13.35	≤16.5
Granulocyte Colony-Stimulating Factor (G-CSF)	**12.38**	≤9.48
Granulocyte-Macrophage Colony-Stimulating Factor (GM-CSF)	2.62	≤4.19

Table S3. Lymphocyte subset counts levels during hospitalization. Blue text in highlights values below the normal range, to facilitate visual distinction

Test Item Name	Test Result	Reference Range
Lym %	99.24	
CD3 %	66.39	50-82
CD4 %	25.67	24-54
CD8 %	39.28	14-41
CD4/CD8	**0.65**	0.7-3.1
CD19 %	16.65	5-21
CD16/CD56 %	16.21	6-38
CD45 cells/μL	410.37	
CD3 cells/μL	**272.43**	723-2737
CD4 cells/μL	**105.34**	404-1612
CD8 cells/μL	**161.19**	220-1129
CD19 cells/μL	**68.31**	80-616
CD16/CD56	**66.51**	84-724

Lym%, Lymphocyte Percentage; **CD3%,** CD3+ T Lymphocyte Percentage; **CD4%,** CD4+ T Helper Cell Percentage; **CD8%,** CD8+ Cytotoxic T Cell Percentage; **CD4/CD8,** CD4/CD8 Ratio; **CD19%,** CD19+ B Lymphocyte Percentage; **CD16/CD56%,** CD16+CD56+ Natural Killer (NK) Cell Percentage; **CD3,** CD3+ T Lymphocyte Absolute Count; **CD4,** CD4+ T Helper Cell Absolute Count; **CD8,** CD8+ Cytotoxic T Cell Absolute Count; **CD19,** CD19+ B Lymphocyte Absolute Count; **CD16/56,** CD16+CD56+ NK Cell Absolute Count.

Table S4. The results of blood and fecal tests of the patient.

Test Item Name	14/11/2023	22/11/2023	30/11/2023	9/12/2023
Routine blood investigations				
WBC (10^9)	3.87	3.54	4.66	4.28
RBC (10^9)	3.24	3.10	3.1	3.12
HGB (g/L)	106	104	105	103
PLT (10^9)	181	112	102	131
NEUT%	69.7	83.3	76.6	67.8
CRP (mg/L)	18	10.6	5.3	5.5
PCT (ng/L)	0.068	-	-	0.066
ALB (g/L)	27.2	38.4	33.6	33.9
TP (g/L)	43.6	53.2	50.3	52.8

PA (g/L)	88	215	287	271
K+ (mmol/L)	3.36	4.08	3.15	3.69
Na+ (mmol/L)	133.2	140	137	138
Fecal examination				
F-RBC	15-20/HPF	NA	NA	NA
F-WBC	per HPF	NA	NA	NA
F-Fungus	NA	NA	NA	NA
Bacterial culture	NA	NA	NA	NA
OB	positive	positive	positive	positive

WBC, white blood cells count; **RBC,** red blood cells count; **HGB,** hemoglobin; **PLT**, platelet count; **CRP,** C-reactive protein; **PCT,** procalcitonin; **NEUT%,** neutrophil percentage**; ALB,** albumin; **TP,** total protein; **PA,** prealbumin; **K,** potassium ions; **NA,** normal range; **OB,** occult blood; **–,** not examined.

Table S5. The results of blood and fecal tests of the patient.

Test Item Name	Test Result	Reference Range
Virological blood investigations		
EB-IgG(CA)	>750	<20
EB-IgG(NA)	102	<20
EB-IgM(EA)	0.02	<1.1
EB-IgM(CA)	4.09	<40
CMV-IgM	0.227	0-1
CMV-IgG	428	0-1
HCMV	<500	<500
EBV-DNA	<500	<500
Fecal viral nucleic acid investigations		
HCMV	<500	<500
EBV-DNA	<500	<500

EB-IgG (CA), Epstein–Barr Virus Capsid Antigen Immunoglobulin G**; EB-IgG (NA),** Epstein–Barr Virus Nuclear Antigen Immunoglobulin G; **EB-IgM (EA),** Epstein–Barr Virus Early Antigen Immunoglobulin M; **EB-IgM (CA)**, Epstein–Barr Virus Capsid Antigen Immunoglobulin M; **CMV-IgM,** Cytomegalovirus Immunoglobulin M; **CMV-IgG,** Cytomegalovirus Immunoglobulin G; **HCMV,** Human Cytomegalovirus; **EBV-DNA,** Epstein–Barr Virus Deoxyribonucleic Acid.

Table S6. Naranjo Adverse Drug Reaction Assessment Scale.

Related Questions	Question Scores			
	Yes	No	Unknow	Scores
1. Are there conclusive reports on Sintilimab-related immune colitis?	+1	0	0	+1

2. Did immune colitis occur after the use of Sintilimab?	+2	-1	0	+2
3. Did Immune colitis resolve after discontinuation of Sintilimab?	+1	0	0	+1
4. Did immune colitis recur after re-use of Sintilimab?	+2	-1	0	0
5. Are there other causes that could cause immune colitis?	-1	+2	0	0
6. Does this adverse reaction recur after the application of placebo?	-1	+2	0	0
7. Does Sintilimab reach a toxic concentration in the blood or other body fluids?	-1	+1	0	0
8. Does Immune colitis worsen with an increase in the dose of Sintilimab or relieve with a decrease in the dose?	+1	0	0	0
9. Has the patient been exposed to similar drugs and had a similar reaction?	+1	0	0	0
10. Is there objective evidence of this adverse reaction with the patient's Immune colitis?	+1	0	0	+1
Total score				6

A total score of ≥ 9 indicates a definite association between the drug and the adverse reaction. A total score between 5 and 8 indicates a high probability of an association between the two. A total score of 1-4 indicates that there may be some association. A total score of ≤ 0 indicates that this association is suspect.

Table S7. Specific therapeutic strategies for ir-colitis in various guidelines.

	NCCN	EMSO	ASCO	SITC	CSCO
**Grade1**	Observation, symptomatic treatment, mesalazine if necessary	Observation	Observation, symptomatic treatment		
**Grade2**	Oral Budesonide; prednisone/methylprednisolone 1mg/kg/d; Biologics; JNK inhibitors	Oral corticosteroids 40-60mg/d	Oral prednisone/methylprednisolone 1mg/kg/d		

**Grade3-4**	Intravenous methylprednisolone 1-2mg/kg/d;<br>Biologics;<br>JNK inhibitors	Intravenous methylprednisolone 1mg/kg/d	Intravenous methylprednisolone 1-2mg/kg/d;<br>Biologics	Intravenous methylprednisolone 1-2mg/kg/d;<br>Biologics

NCCN: National Comprehensive Cancer Network; EMSO: European Society of Medical Oncology; ASCO: American Society of Clinical Oncology; SITC: American Society for Immunotherapy of Cancer; CSCO: Chinese Society of Clinical Oncology.

Supplementary Figures

Figure S1. PCT levels during hospitalization.

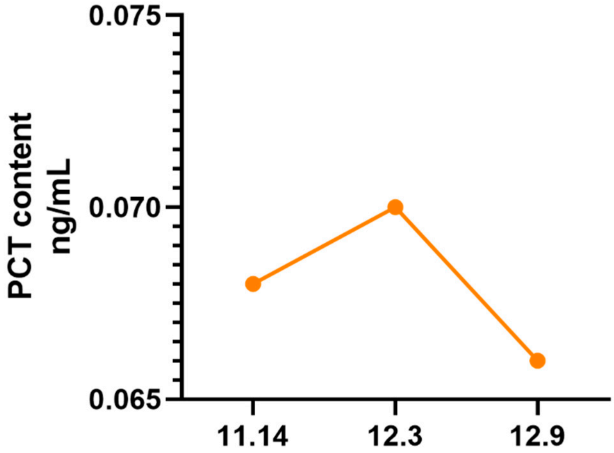

The patient's procalcitonin (PCT) levels remained within the normal range during hospitalization.

Figure S2. Serum ALB, TP, and PA levels during hospitalization and at 18-month follow-up.

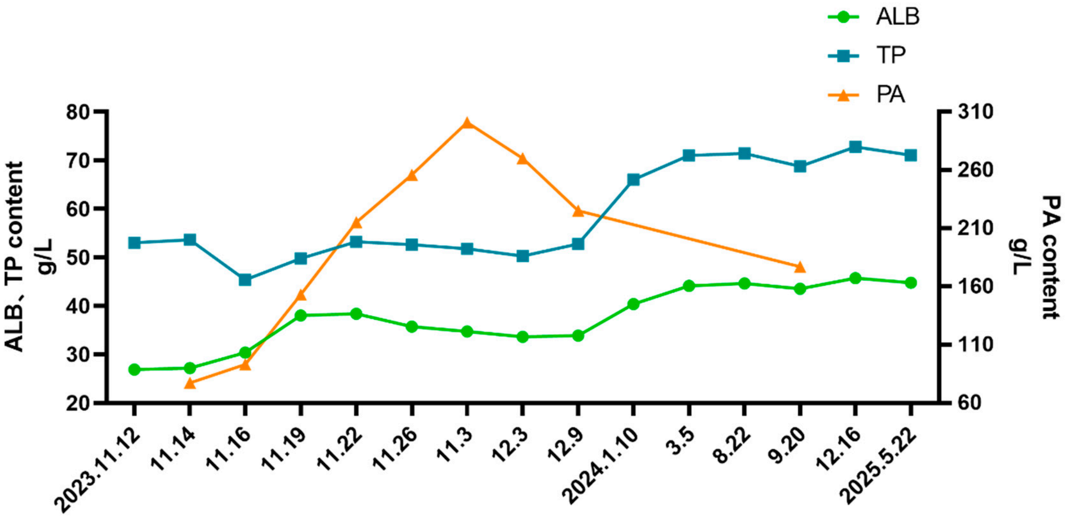

During hospitalization, the patient's levels of prealbumin (PA), albumin (ALB), and total protein (TP) were below the normal range, indicating poor nutritional status. After disease control, these indicators gradually increased, suggesting improved nutritional status and favorable disease recovery.

Figure S3. Serum Na+, K+ levels during hospitalization and at 18-month follow-up.

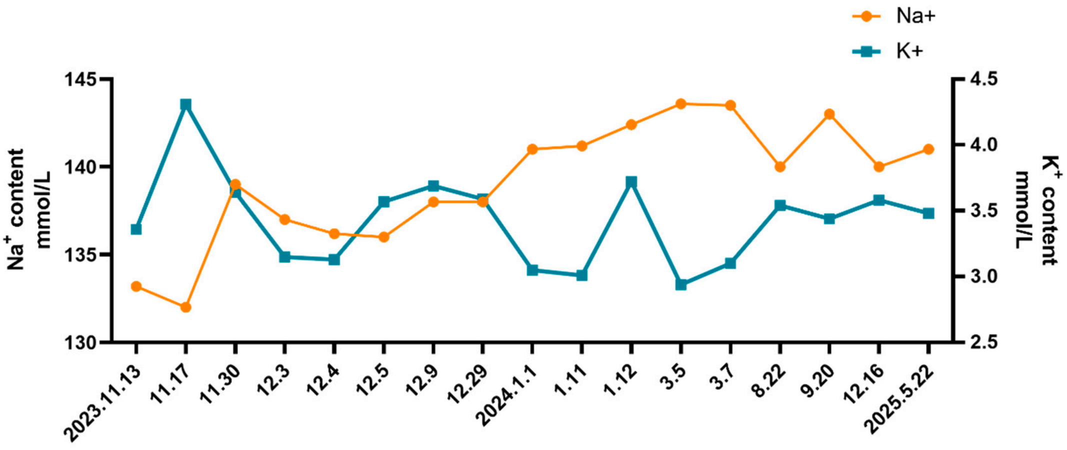

At admission, the patient exhibited significant fluctuations in electrolyte levels, reflecting active disease. Following disease control, electrolyte levels gradually stabilized.
